# EvoCor: a platform for predicting functionally related genes using phylogenetic and expression profiles

**DOI:** 10.1093/nar/gku442

**Published:** 2014-05-21

**Authors:** W. James Dittmar, Lauren McIver, Pawel Michalak, Harold R. Garner, Gregorio Valdez

**Affiliations:** 1 Virginia Tech Carilion School of Medicine, Roanoke, VA 24016, USA; 2 Virginia Bioinformatics Institute, Virginia Tech, Blacksburg, VA 24061, USA; 3 Virginia Tech Carilion Research Institute, Roanoke, VA 24016, USA; Department of Biological Sciences, Virginia Tech, Blacksburg, VA 24061, USA

## Abstract

The wealth of publicly available gene expression and genomic data provides unique opportunities for computational inference to discover groups of genes that function to control specific cellular processes. Such genes are likely to have co-evolved and be expressed in the same tissues and cells. Unfortunately, the expertise and computational resources required to compare tens of genomes and gene expression data sets make this type of analysis difficult for the average end-user. Here, we describe the implementation of a web server that predicts genes involved in affecting specific cellular processes together with a gene of interest. We termed the server ‘EvoCor’, to denote that it detects functional relationships among genes through *ev*olutionary analysis and gene expression *cor*relation. This web server integrates profiles of sequence divergence derived by a Hidden Markov Model (HMM) and tissue-wide gene expression patterns to determine putative functional linkages between pairs of genes. This server is easy to use and freely available at http://pilot-hmm.vbi.vt.edu/.

## INTRODUCTION

The human genome contains over 21,000 protein-coding genes (1), and yet contemporary scientific inquiry tends to devote a disproportionate amount of time to studying the function of a few genes at a time (2). This focus is in large part due to the time and resources required to identify additional candidate genes using conventional biochemical and molecular methods. The recent explosion of genomic and expression datasets has provided opportunities to develop computational tools that can quickly generate lists of candidate genes that could play key roles together with a query gene in driving a complex, yet specific cellular process. These recently developed computational methods take advantage of sequence information and gene expression patterns and are based on calculating similarity profiles using Hamming distance (3); mutual information using co-occurrence (4); maximum likelihood branch-length model-based (5); and a combination of phylogenetic and co-expression analyses (6). Although these approaches have been successfully used to identify functional relationships between a gene of interest and other eukaryotic genes (7), the computational resources and expertise needed to implement these methods make them unavailable to most biologists.

In this paper, we describe a web server that incorporates phylogenetic profiles and gene expression patterns to predict functional relationships between a gene of interest and all known eukaryotic genes. We named this server EvoCor because it employs *ev*olutionary phylogenetics and gene expression data to predict genes functionally *cor*related with an input gene. Using *Shadowfax*, a 64 node computing cluster with 2× 2.9 GHz 6-core processors, we pre-calculated all possible pairwise relationships for all protein coding genes in the human genome, constructed a binary vector to represent the evolutionary history of each gene using 182 different eukaryotic genomes. We also used datasets of expression profiles from human and mouse tissues and cell lines to infer gene expression pattern associations. This server is user-friendly and the data generated easy to interpret, allowing end-users to freely and quickly obtain lists of candidate genes that may play key roles in specific biological processes.

## MATERIALS AND METHODS

### Construction of presence/absence trait vector

**Figure 1.**
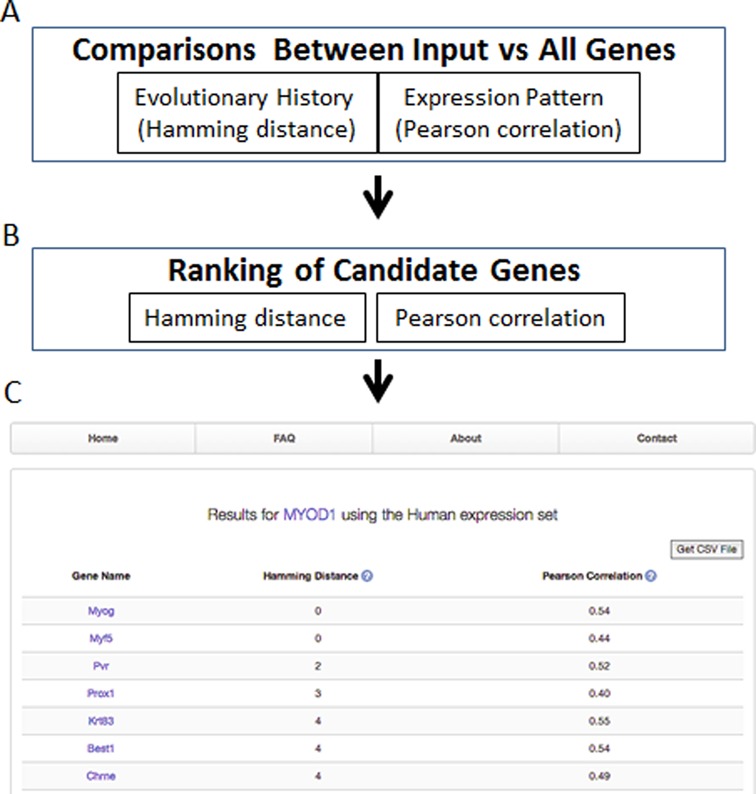
Workflow of an EvoCor analysis. The evolutionary history and expression pattern is first compared between the input and all genes (**A**). EvoCor then utilizes this information to rank genes based on their similar evolutionary history and expression pattern to the input gene (**B**) and generates a list of functionally related genes (**C**).

To represent the evolutionary history of each gene, we constructed a binary vector (3) of length 182, which represents the total number of Eukaryotic species in the NCBI database (Release 57) as of this writing. Each point in the vector encodes a one or a zero, which indicates whether a sequence homolog can be found in that species. In contrast to previous methods, we employ a Hidden Markov Model (HMM) Profile search using HMMER3 (8) to determine sequence homologs. This method allows for improved detection of remote homologs (9) because it does not generalize site-specific transition rates for insertions and deletions. We used an empirically derived cutoff and defined a gene to be ‘present’ in a given species if the HHMER3 search yields any sequences in that organism that contains at least one domain with an expected (*E*) value of <1E−7. We then used these matrices to calculate the pairwise Hamming distance between the gene of interest and every other protein-coding gene in the human genome. We hypothesized that genes that function in the same cellular process are under similar evolutionary selection pressures. These genes are therefore likely to show a correlated pattern of sequence divergence (Figure 1A-C).

### Calculating the pearson correlation coefficient

For each gene pair, we calculate the Pearson correlation coefficient based on a tissue-wide atlas of gene expression data from a range of human (NCBI GSE1133) (10,11) as well as mouse (NCBI GSE10246) (11) using the WGCNA package in *R* (12). The NCBI GSE11233 and GSE10246 atlas contain the transcriptome of a variety of normal tissues and cell lines obtained from humans and mice, respectively. For both expression sets, the raw Affymetrix data was background corrected, log2 transformed and quantile normalized, as per the Robust Multi-array Average (RMA) algorithm (13). EvoCor utilizes this dataset to identify genes expressed in the same tissues as the input gene and this is represented as the Pearson coefficient in the output of the server (Figure 1B).

### Ranking candidate genes

EvoCor ranks candidate genes first based on their evolutionary history (Hamming distance) followed by their expression correlation (Pearson correlation). Increasing Hamming distance values (0 to 182) indicate evolutionary divergence between the candidate and query gene. Thus, candidate genes with lower Hamming distance values appear at the top of the list. EvoCor then uses expression data to refine the list. First, genes with a low expression correlation (<0.2) are not included in the output list. It then ranks candidate genes with the same Hamming distance based on their Pearson correlation, with candidates most likely co-expressed with the query gene occupying the top spots. Our rational for this ranking scheme is that the evolutionary history best predicts genes with similar function or involved in the same cellular process whereas expression analysis helps refine the list, reduce the number of false positive and assign a higher rank to candidate genes most likely to be present in the same cells or tissues as the query gene.

## RESULTS

### Evaluation of EvoCor

**Figure 2.**
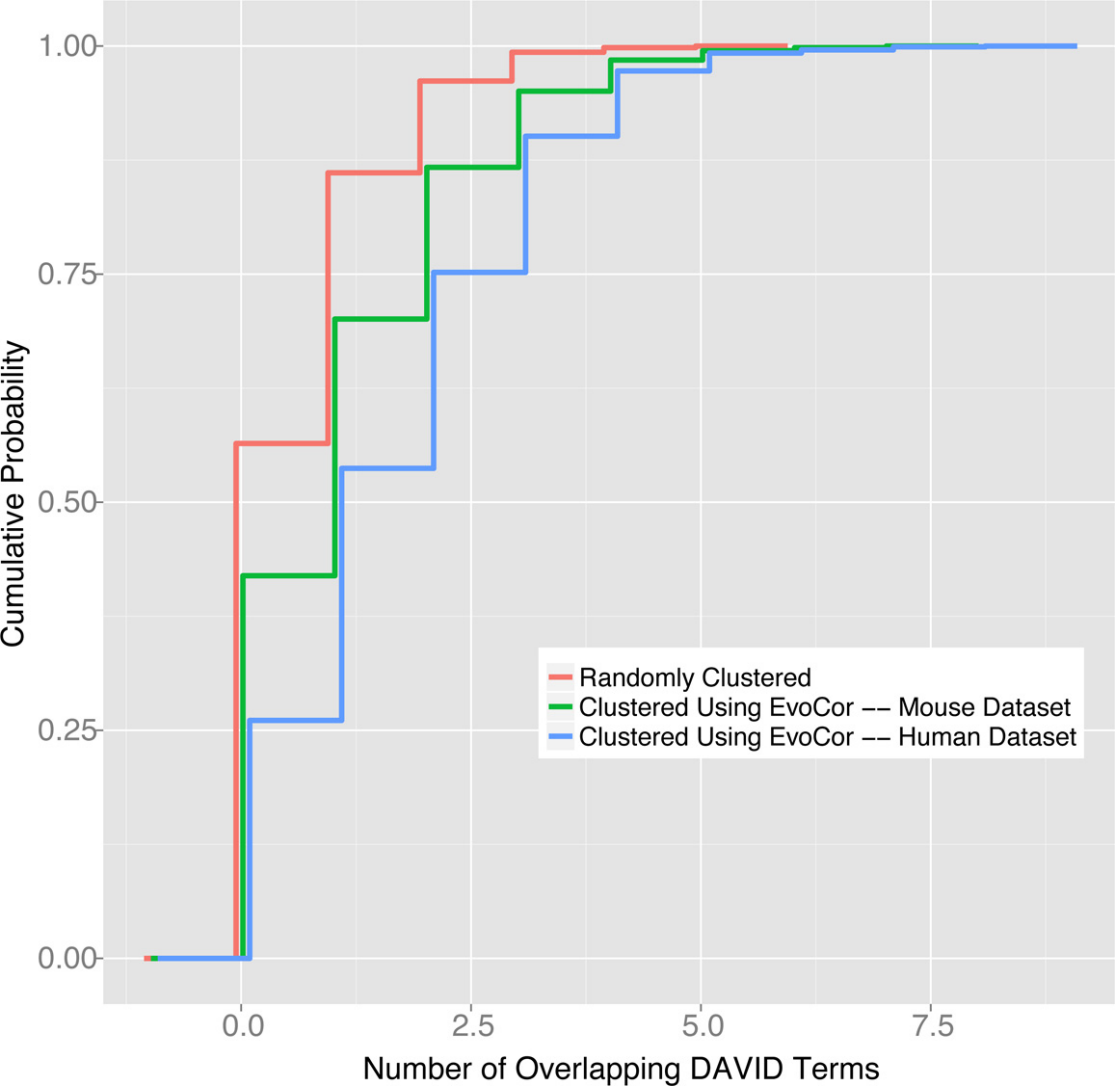
DAVID was used to evaluate EvoCor ability to predict genes with similar biological functions. The fraction of DAVID's key word overlap was determined for gene sets clustered using EvoCor (green and blue lines) and clustered randomly (red line). The percentage of DAVID's overlapping terms is significantly higher in the gene sets generated using EvoCor with the human gene expression set (blue line) as well as the mouse expression set (green line) compared to randomly generated gene sets (red line). For the human expression dataset versus *P*-value random the *P*-value = 2.20e-16 and *D*-value = .3286. For the mouse expression dataset versus random the *P*-value = 2.22E−16 and *D*-value = 0.1664.

Before deploying EvoCor, we tested its ability to predict functionally related genes. For this, we used the programmatic interface for the Database for Annotation, Visualization and Integrated Discovery (DAVID) (14) to examine the ability of EvoCor to recover functionally related groups of genes based on phylogenetics and expression profiling. Our hypothesis is that EvoCor will recover more functionally related groups of genes than would be expected by random association. To test this hypothesis, we first generated a set of 2500 randomly selected genes from the human genome and gathered the top 125 results for each gene generated by EvoCor. We ordered our results first by Hamming distance, then by the Pearson correlation. We included only those results with a non-zero Pearson Correlation coefficient, thus excluding genes not represented by the microarrays. For the control group, we randomized the list of 312 500 genes (125 results for 2500 genes), and repeated the same procedure. We then randomized the order of each 125 gene result set for each group and divided each result set into two separate lists. The two separate lists for each result set were run against DAVID's Functional Annotation Clustering with default settings, which include Gene Ontology (GO) terms and other functional themes. We quantified the number of overlapping terms within the result set of each group. We expected EvoCor to return a higher percentage of overlapping terms within the top 125 results than would be expected by chance alone. We compared the distributions of results for each of these groups using a two-sided Kolmogorov-Smirnov test and obtained a *D*-value of .3286 (*P*-value of 2.12E−16) using the human expression dataset and a *D*-value of 0.1664 (*P*-value of 2.22E−16) using the mouse expression dataset, suggesting that EvoCor is able to detect functionally related groups of genes (Figure 2).

To verify the assumption that the addition of expression data to the evolutionary information improves the performance of EvoCor, we performed an initial validation test. We compared the pairwise GO term overlap between 2500 random query genes and their corresponding top 125 results using evolutionary data alone as well as expression data in addition to evolutionary data using the GoSemSim package in *R* (15). We find a very significant improvement in the overlap when both phylogenetic and expression profiles are used relative to phylogenetic profile alone (Wilcoxon rank-sum test, from *W* = 4576217071 to *W* = 4668457224, *P* = 1.291E−13). We therefore conclude that the addition of expression data to phylogenetic profiling improves the ability to predict functional relationships.

As an illustrative example, EvoCor was used to retrieve a list of genes predicted to co-evolve and be co-expressed with a well-characterized gene, myogenic differentiation 1 (*Myod1*). *Myod1* belongs to a family of transcription factors known to regulate muscle biogenesis that includes *myogenin (Myog), Myf5* and *Myf6* (16). As shown in Figure 1C, EvoCor predicts that *Myog* and *Myf5* are the most likely candidate genes to be involved with *Myod1* in muscle biogenesis using the human expression dataset. All three family members, *Myog, Myf5 and Myf6*, are the top candidate genes using the mouse expression dataset (not shown). In fact, the majority (75 using the human and 99 using the mouse expression dataset) of the top 125 predicted genes have been demonstrated to play essential roles in muscle tissue, with the other genes having features that could make them attractive candidates for regulating different aspects of muscle physiology. Furthermore, a large number of the top candidate genes or close family members appear in both lists of candidate genes generated for *Myod1* using the human and mouse expression datasets. Although there are clear differences between the human and mouse dataset, these are likely due to differences in the microarrays and the types of tissues and cells examined. For example, the mouse array contains the transcriptome of skeletal muscles and of cultured muscles derived from C2C12 cells, a myogenic cell line that can be transformed into skeletal muscles. However, the mouse expression dataset lacks the transcriptomes of cardiac and smooth muscle cells, which are well represented in the human expression dataset. Because of sequence divergence and other physiologic differences between mouse and human, the probes used in the two different microarrays are likely to generate different signals (17). Thus, many genes that function alongside *Myod1* in the proliferation and differentiation of cardiac and smooth muscles would naturally be missing from the mouse array. Irrespective, this example demonstrates the power of using EvoCor to identify candidate genes and develop hypothesis for further experimentation.

### Comparison to existing methods

We improve upon and differentiate from the major existing methods that search for functional linkages (3,18–20) in the following ways: first, we use Hidden Markov Model Profiles to search for sequence homologs, rather than BlastP similarity searches; secondly, we use all fully sequenced Eukaryotic genomes that are currently available; third, we implement a simple interface that will facilitate its use amongst biologists; and finally, we supplement phylogenetic inference with expression data to decrease the high rate of false positives. These features allow EvoCor to make unbiased predictions on genes that may function along a complex cellular process to elicit a very specific biological outcome. Together with other tools, including HumanNet, STRING and Predictome, which rely on prior knowledge of the function and interaction of proteins, EvoCor will help end-users identify novel candidate genes quickly and free.

*Conflict of interest statement*. None declared.
